# Characteristics of *Aedes aegypti* adult mosquitoes in rural and urban areas of western and coastal Kenya

**DOI:** 10.1371/journal.pone.0189971

**Published:** 2017-12-19

**Authors:** Bryson Alberto Ndenga, Francis Maluki Mutuku, Harun Njenga Ngugi, Joel Omari Mbakaya, Peter Aswani, Peter Siema Musunzaji, John Vulule, Dunstan Mukoko, Uriel Kitron, Angelle Desiree LaBeaud

**Affiliations:** 1 Centre for Global Health Research, Kenya Medical Research Institute, Kisumu, Kenya; 2 Department of Environment and Health Sciences, Technical University of Mombasa, Mombasa, Kenya; 3 Department of Biological Sciences, Chuka University, Chuka, Kenya; 4 Department of Zoology, University of Nairobi, Nairobi, Kenya; 5 Vector Borne Disease Control Unit, Ministry of Health, Msambweni, Kenya; 6 Centre for Infectious and Parasitic Diseases Control Research, Kenya Medical Research Institute, Busia, Kenya; 7 Vector Borne Disease Control Unit, Ministry of Health, Nairobi, Kenya; 8 Department of Environmental Sciences, Emory University, Atlanta, Georgia, United States of America; 9 Department of Pediatrics, Stanford University, Stanford, California, United States of America; Institut Pasteur, FRANCE

## Abstract

*Aedes aegypti* is the main vector for yellow fever, dengue, chikungunya and Zika viruses. Recent outbreaks of dengue and chikungunya have been reported in Kenya. Presence and abundance of this vector is associated with the risk for the occurrence and transmission of these diseases. This study aimed to characterize the presence and abundance of *Ae*. *aegypti* adult mosquitoes from rural and urban sites in western and coastal regions of Kenya. Presence and abundance of *Ae*. *aegypti* adult mosquitoes were determined indoors and outdoors in two western (urban Kisumu and rural Chulaimbo) and two coastal (urban Ukunda and rural Msambweni) sites in Kenya. Sampling was performed using quarterly human landing catches, monthly Prokopack automated aspirators and monthly Biogents-sentinel traps. A total of 2,229 adult *Ae*. *aegypti* mosquitoes were collected: 785 (35.2%) by human landing catches, 459 (20.6%) by Prokopack aspiration and 985 (44.2%) by Biogents-sentinel traps. About three times as many *Ae*. *aegypti* mosquitoes were collected in urban than rural sites (1,650 versus 579). Comparable numbers were collected in western (1,196) and coastal (1,033) sites. Over 80% were collected outdoors through human landing catches and Prokopack aspiration. The probability of collecting *Ae*. *aegypti* mosquitoes by human landing catches was significantly higher in the afternoon than morning hours (P<0.001), outdoors than indoors (P<0.001) and in urban than rural sites (P = 0.008). Significantly more *Ae*. *aegypti* mosquitoes were collected using Prokopack aspiration outdoors than indoors (P<0.001) and in urban than rural areas (P<0.001). Significantly more mosquitoes were collected using Biogents-sentinel traps in urban than rural areas (P = 0.008) and in western than coastal sites (P = 0.006). The probability of exposure to *Ae*. *aegypti* bites was highest in urban areas, outdoors and in the afternoon hours. These characteristics have major implications for the possible transmission of arboviral diseases and for the planning of surveillance and control programs.

## Introduction

*Aedes aegypti* is an important vector of arboviruses, which include yellow fever, dengue, chikungunya and Zika viruses [1–4]. This vector originated in Africa [5], spread to other continents through trade [6] and now is distributed worldwide [7]. Originally a tree-hole forest mosquito, its larvae have adapted to develop in human made containers in the urban environment [6]. *Aedes aegypti* feeds preferentially and frequently on humans [8,9]. Dispersal of its females is mainly determined by the availability of oviposition sites [10]. In most cases *Ae*. *aegypti* mosquitoes do not move from houses where they have been released and for those few that move, their dispersal distances may not exceed 200 meters [11]. Adult *Ae*. *aegypti* mosquitoes feed often and multiple feedings are common [12]. An increase in the number of *Ae*. *aegypti* adult mosquitoes has been associated with an increased probability of the occurrence of arboviral diseases [13,14]. *Aedes aegypti* mosquitoes show a range of color and pattern of scaling, choice for blood meal source, egg dormancy period, choice for oviposition/larval sites, aquatic development time periods and competence to vector viruses [6].

*Aedes aegypti* is widespread in Kenya, although it has not been studied extensively, and its distribution is not uniform, being most common in the lowlands [15]. It exists in two forms, domestic and sylvatic, which have been found in sympatry along the Kenyan coast at Rabai [6]. The domestic form, *Ae*. *aegypti aegypti* is light colored and the sylvatic form, *Ae*. *aegypti formosus* is dark. *Aedes aegypti formosus* occurs in vegetated ecosystems and has been documented in western Kenya near Kisumu City and in Kakamega Forest [16]. In a study in the coastal town of Malindi, *Ae*. *aegypti* mosquitoes were found inside houses [17], while in a recent study in the major coastal city of Mombasa most *Ae*. *aegypti* were found outdoors [18].

Transmission of arboviruses by *Ae*. *aegypti* occurs across Kenya [19,20]. Dengue and chikungunya infections continue to occur in areas where no epidemics have been reported as in western Kenya and between known epidemics in the coastal and north-eastern areas of the country [20–27]. Recent outbreaks reported in Kenya include dengue in Mombasa in 2013 [28] and in 2017 [29] and chikungunya in Mandera in 2016 [30]. As part of a larger on-going eco-epidemiological study (NIH R01 AI102918), this study aimed to characterize the presence and abundance of *Ae*. *aegypti* adult mosquitoes from rural and urban sites in western and coastal regions of Kenya.

## Materials and methods

### Study site

**Fig 1:**
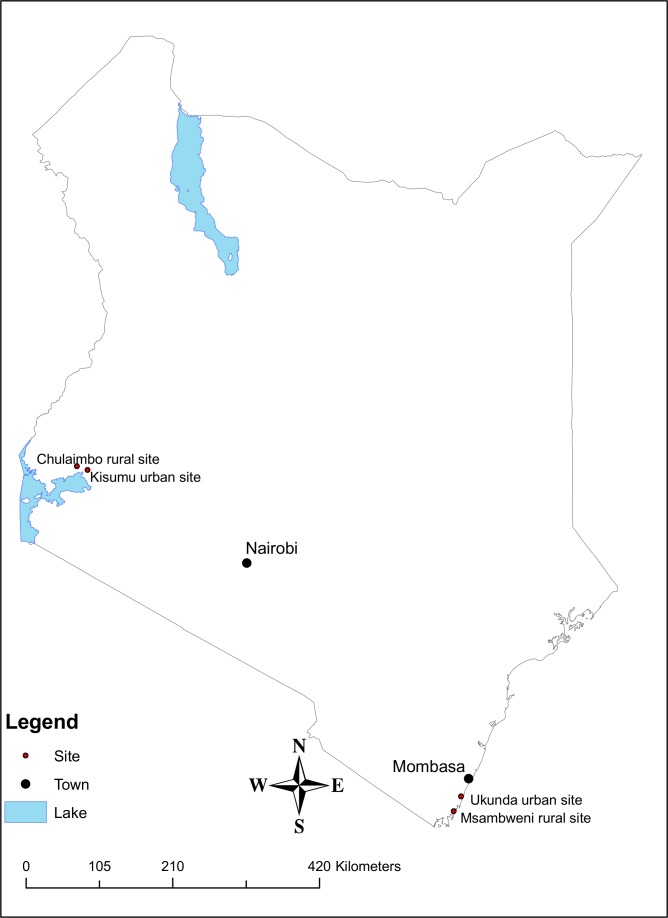
Study site map. A map showing the four study areas: Kisumu, Chulaimbo, Ukunda and Msambweni.

This study was conducted in four sites in Kenya: two western sites, Kisumu urban site (0°5′15.22478′′S, 34°46′22.3284′′E, altitude 1186 meters above sea level (m.a.s.l.)) and Chulaimbo rural site (0°2′17.2500′′S, 34°38′18.1998′′E, altitude 1372 m.a.s.l.) in Kisumu County and two coastal sites, Ukunda urban site (4°16′38.8992″S, 39°34′9.0012″E, altitude 23 m.a.s.l.) and Msambweni rural site (4°27′58.4382″S, 39°28′17.8716″E, altitude 20 m.a.s.l.) in Kwale County (Fig 1).

Urban describes geographic areas that are located inside towns and cities whereas rural describes geographic areas that are located outside towns and cities, usually less developed with significant land cover under agriculture and/or natural vegetation. Kisumu is the third largest city in Kenya located near the shores of Lake Victoria. It is the headquarters of Kisumu County and a business hub for the East African Community. Chulaimbo area is located 19 kilometers from Kisumu along the Kisumu–Busia Road. Ukunda is an emerging urban centre located 30 kilometers south of Mombasa in Kwale County along the Indian Ocean coastline. Diani Beach, a famous tourist destination, is located one kilometer from Ukunda Town. Msambweni is also in Kwale County, near the shores of the Indian Ocean. It is located 30 kilometers south of Ukunda. The climate in both regions is tropical with bimodal rainfall: long rains from March to June and short rains from August to October. Common mosquito-borne diseases in these counties include arboviruses, malaria and lymphatic filariasis. Sampling for *Ae*. *aegypti* mosquitoes in each of the four sites was done in a selected area of 1.5 x 1.0 kilometers.

### Human landing catches (HLC)

Two homesteads were selected in each of the four sites for sampling of *Ae*. *aegypti* mosquitoes using HLC. Adults (≥18 years old), who resided within the sampling area (assumed to be exposed to similar possible mosquito bites) and who provided written informed consent volunteered to catch mosquitoes by HLC. They were all trained before they started the catching of mosquitoes. Two teams of two people each sampled indoors and outdoors (~3–5 meters from the house). “Outdoors” describes any place outside the doors of the house selected for sampling, round and about it but within the homestead. “Indoors” describes the space within the door(s) of the selected house. Both collectors sat on chairs, with one exposing the legs, and the other collecting mosquitoes landing on the partner’s legs. The team members changed roles hourly. Sampling was conducted in June, September, December and March starting from June 2014 to June 2016 from 5:30 am to 11:30 am in the morning and then from 3:30 pm to 7:30 pm in the afternoon. Mosquitoes collected each hour were put in a pre-labelled plastic cup and provided with 10% sugar solution on cotton wool. All labelled plastic-cups were put in a cooler box with ice pack and then transported to the insectaries at Kenya Medical Research Institute, Centre for Global Health Research station at Kisian in Kisumu County for western sites and at the Vector Borne Disease Control Unit in Msambweni County Hospital, Kwale County for coastal sites. At the end of every HLC exercise, all HLC volunteers were examined for malaria infection by the project clinical officer in each of the four sites. Those who tested malaria positive were treated according to the Kenyan Ministry of Health guidelines. The project paid for all their malaria testing and treatment costs. The project clinical officers checked these volunteers for possible dengue and chikungunya symptoms.

### Prokopack automated aspirators

Twenty houses were randomly selected in each of the four sites and sampled indoors and outdoors monthly for *Ae*. *aegypti* mosquitoes using Prokopack aspirators [31] (The Prokopack automated aspirators were constructed with local materials under the guidance of one of the inventors). Sampling by a pair of trained entomology team members was conducted simultaneously for 20 minutes both indoors and outdoors. Two plastic cups were used for each house, one indoors and the other outdoors. All labelled plastic-cups were put in a cooler box with ice pack and transported as described above.

### Biogents-sentinel traps (BG)

One house was selected in each of the four sites for the sampling of *Ae*. *aegypti* mosquitoes using Biogents (BG)-traps (Biogents AG Weissenburgstr 22 93055 Regensburg, Germany). The BG traps were placed in secure verandas and were baited with carbon dioxide (CO_2_). The CO_2_ was produced from a mixture of 17.5 grams yeast (Angel Yeast (Egypt) Co. Ltd.), 250 grams sugar in 2 liters of water. In August 2015, these amounts were increased to 35 grams yeast, 500 grams sugar in 5 liters of water in order to produce more CO_2_. This mixture was replaced on the third day after setting up the experiment. The BG trap was set to sample mosquitoes monthly for five consecutive days. Every day at about midday, the battery was replaced with a charged one. Trapped mosquitoes in the collection net were put in a cooler box daily with ice pack and then transported as described above.

### Mosquito identification

In the insectaries, all mosquitoes were killed by placing them at -20 degrees for 15 minutes. They were then sorted by genus (*Aedes*, *Anopheles*, *Culex*, *Mansonia* or *Toxorhynchites*) and sex. Females were further sorted according to their blood-feeding stages as unfed, blood-fed, half-gravid or gravid. *Aedes* mosquitoes were further identified to species using identification keys [32,33] as either *Ae*. *aegypti* or *Ae*. *simpsoni*. However, we could not further identify *Ae*. *aegypti* mosquitoes either as *Ae*. *aegypti aegypti* or *Ae*. *aegypti formosus*.

### Rainfall

One rain gauge (HOBO® Onset data loggers, Onset Computer Corporation 470 Bourne, MA, USA), was installed at a central place in each of the four sites to collect daily rainfall: Chulaimbo County Hospital (Chulaimbo); Jaramogi Oginga Odinga Teaching and Referral Hospital (Kisumu); Msambweni County Hospital (Msambweni) and Diani Health Centre (Ukunda). Data from these rain gauges were downloaded monthly.

### Ethical considerations

This study was approved by both Stanford University Institutional Review Board (Protocol ID 31488 and IRB Number 6208) and Kenya Medical Research Institute National Ethical Review Committee (SSC No. 2611). Meetings were held in each Sub-Location at all the four sites with local government administrators (county commissioners, chiefs and assistant chiefs) and the local residents to introduce the research study and staff to the public. All study participants in this study were adults (≥18 years old). A written and signed consent was obtained from all adults who volunteered to participate in HLC before they were trained and started sampling for mosquitoes. A copy of the signed consent form was given to each of the HLC volunteer and another kept in a locked cabinet with restricted access in offices at Kenya Medical Research Institute, Centre for Global Health Research station at Kisian in Kisumu County and at the Vector Borne Disease Control Unit in Msambweni County Hospital, Kwale County. Verbal consent was obtained from household heads to sample mosquitoes in their houses and compounds.

### Data analysis

Monthly totals of *Ae*. *aegypti* were used in all analyses for the mosquitoes collected by Prokopack aspirators and BG traps whereas quarterly totals were used for those collected by HLC. Statistical differences in the monthly densities of *Ae*. *aegypti* mosquitoes in study sites (Chulaimbo/Kisumu/Msambweni/Ukunda), rural/urban areas, western/coastal sites, indoors/outdoors, rainfall and different periods of sampling in the morning/afternoon (for HLC) were performed using generalized estimating equations (GEE) on count data that were fitted with a negative binomial distribution with a log link function. Univariate analysis was done for each of the mosquito sampling methods separately. Parameters with P≤0.25 in the univariate analysis were included in multivariate analysis. Any parameter that was set to zero because it was redundant was omitted from the final multivariate analysis. Prokopack aspiration and HLC data collected in the same period, from 1^st^ June 2014 to 30^th^ June 2016, were used in this analysis. BG data used in the analysis were collected from 1^st^ May 2015 to 30^th^ June 2016. Data were analysed using Statistical Package for Social Sciences (SPSS) Version 21.

## Results

### Human landing catches (HLC)

**Fig 2:**
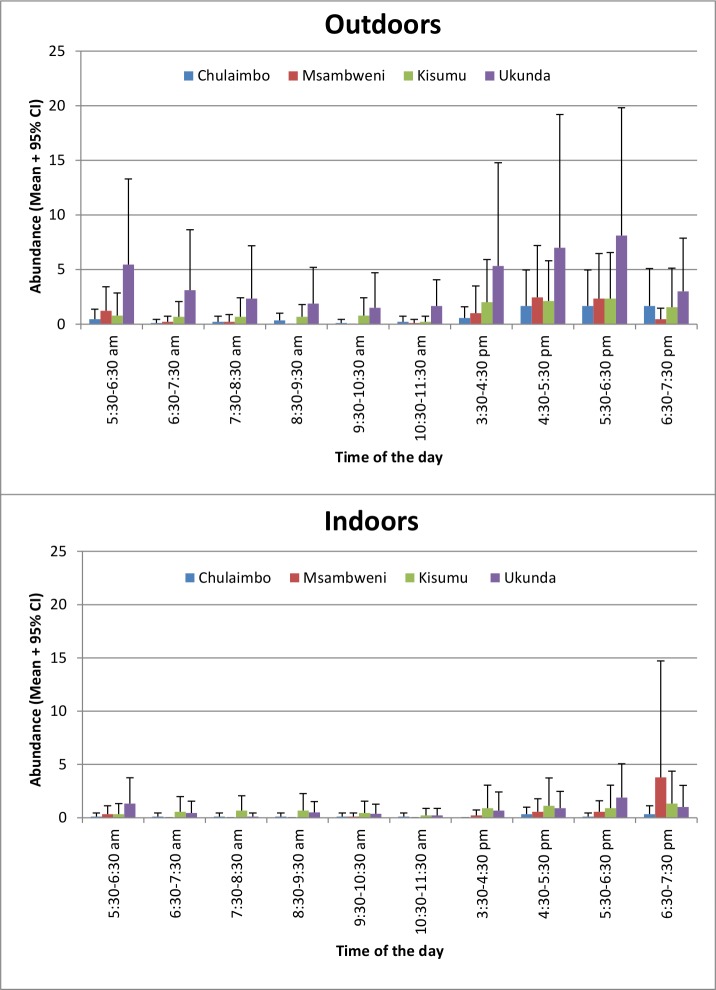
Mean numbers + 95% confidence interval (CI) of *Aedes aegypti* mosquitoes collected hourly by human landing catches.

A total of 785 *Ae*. *aegypti* mosquitoes were collected by HLC: 193 indoors and 592 outdoors and 198 in rural sites and 587 in urban sites. The number of *Ae*. *aegypti* mosquitoes collected varied among the hours, sites and indoors/outdoors (Fig 2). Period (morning/afternoon) hours (P≤0.001), collection (indoors/outdoors) (P≤0.001), site (Chulaimbo/Kisumu/Msambweni/Ukunda) (P≤0.001) and place (rural/urban) (P = 0.002) were statistically significant by univariate analysis whereas region (western/coastal) (P = 0.071) and rainfall (P = 0.905) were not significant. The chances of finding *Ae*. *aegypti* mosquitoes by HLC were significantly higher in the afternoon hours than in the morning, outdoors than indoors and in the urban than rural areas (Table 1).

**Table 1:** Parameters associated with the abundance of *Aedes aegypti* mosquitoes collected by human landing catches, Prokopack aspirators and Bio-Gents sentinel traps.

Method	Parameter	Occasions (N)	Mean (95% CI)	Odds Ratio (95% CI)	P-value
**HLC**	**Period**				
	Afternoon	290	1.8 (1.4–2.2)	3.0 (1.7–5.4)	<0.001
	Morning	430	0.6 (0.5–0.8)	1.0	
	**Collection**				
	Outdoors	360	1.6 (1.3–2.0)	2.7 (1.8–4.2)	<0.001
	Indoors	360	0.5 (0.3–0.8)	1.0	
	**Place**				
	Urban	360	1.6 (1.3–2.0)	2.9 (1.3–6.3)	0.008
	Rural	360	0.6 (0.3–0.8)	1.0	
	**Region**				
	Coastal	360	1.5 (1.1–1.9)	2.0 (0.9–4.4)	0.087
	Western	360	0.7 (0.5–0.9)	1.0	
**Prokopack**	**Collection**				
	Outdoors	100	4.1 (3.1–5.1)	8.0 (5.7–11.3)	<0.001
	Indoors	100	0.5 (0.3–0.7)	1.0	
	**Place**				
	Urban	100	3.5 (2.5–4.5)	3.4 (2.5–4.8)	<0.001
	Rural	100	1.1 (0.7–1.6)	1.0	
	**Rainfall**	200	107.9 (94.6–121.1)	1.0 (1.0–1.0)	0.121
**BG**	**Place**				
	Urban	28	25.5 (15.7–35.3)	1.8 (1.2–2.9)	0.008
	Rural	28	9.7 (6.8–12.6)	1.0	
	**Region**				
	Coastal	28	10.4 (7.1–13.8)	0.5 (0.3–0.8)	0.006
	Western	28	24.8 (15.0–34.5)	1.0	
	**Rainfall**	56	119.1 (89.8–148.3)	1.0 (1.0–1.0)	0.046

### Prokopack automated aspirators

**Fig 3:**
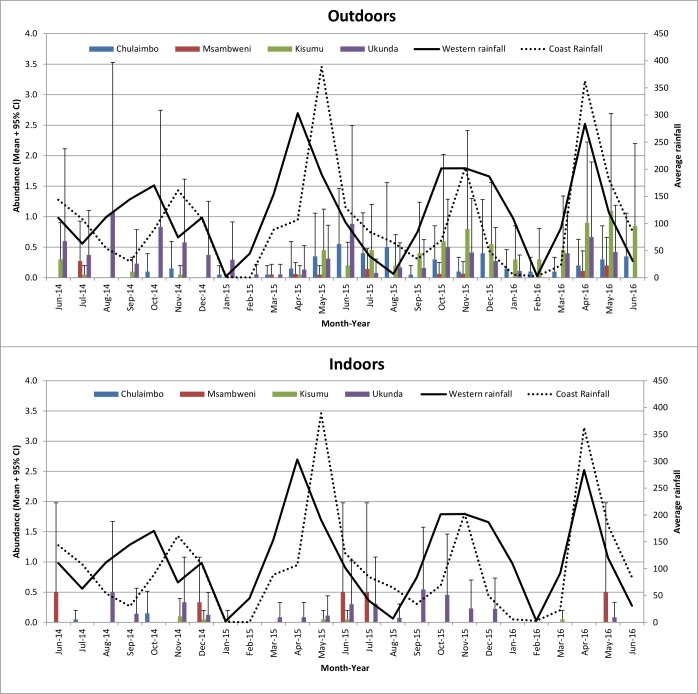
Mean numbers + 95% CI of *Aedes aegypti* mosquitoes collected by Prokopack aspirators.

A total of 459 *Ae*. *aegypti* mosquitoes were collected by Prokopack aspirators: 52 indoors and 407 outdoors and 110 in rural sites and 349 in the urban sites. The number of *Ae*. *aegypti* mosquitoes collected varied by month, indoors/outdoors and among the sites (Fig 3). Collection (indoors/outdoors) (P≤0.001), site (Chulaimbo/Kisumu/Msambweni/Ukunda) (P≤0.001) and place (rural/urban) (P≤0.001) were statistically significant by univariate analysis whereas region (western/coastal) (P = 0.295) and rainfall (P = 0.190) were not significant. The chances of finding *Ae*. *aegypti* mosquitoes by Prokopack aspirators were significantly higher outdoors than indoors and in the urban than the rural areas (Table 1).

### Biogents-sentinel traps (BG)

**Fig 4:**
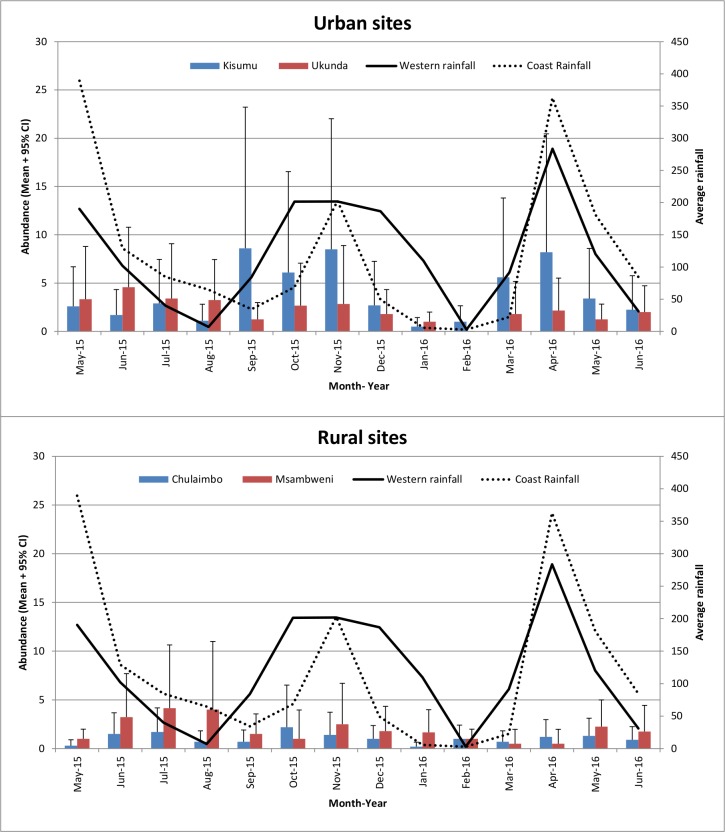
Mean numbers + 95% CI of *Aedes aegypti* mosquitoes collected by Bio-Gents sentinel trap.

A total of 985 *Ae*. *aegypti* mosquitoes were collected using BG-sentinel traps: 271 in rural sites and 714 in urban sites. The number of *Ae*. *aegypti* mosquitoes collected varied by month, indoors/outdoors and among the sites (Fig 4). Site (Chulaimbo/Kisumu/Msambweni/Ukunda) (P≤0.001), place (rural/urban) (P≤0.001), region (western/coastal) (P = 0.001) and rainfall P≤0.001) were statistically significant by univariate analysis. The chances of finding *Ae*. *aegypti* mosquitoes by BG traps were significantly higher in the urban than the rural areas, in western than coastal sites and significantly increased with increase in rainfall (Table 1).

### Mosquitoes collected

A total of 2,229 *Ae*. *aegypti* mosquitoes were collected using all the three methods: 579 in the rural sites and 1,650 in the urban sites. The total number of *Ae*. *aegypti* mosquitoes collected in the urban sites was 2.8 times that collected in the rural sites and comparable between the western (1,196) and coastal (1,033) sites. Out of the 1,244 *Ae*. *aegypti* mosquitoes that were collected by HLC and Prokopack methods, 80.3% (999/1,244) were collected outdoors. Out of the 459 *Ae*. *aegypti* mosquitoes collected by the Prokopack method, 76.7% (352) were females, of which 68.0% (312) were unfed, 5.0% (23) were blood-fed, 1.1% (6) were half-gravid and 2.6% (12) were gravid. A total of 39,172 other mosquitoes were collected: HLC (3,461), Prokopack (19,640) and BG (16,071). They included: 68 *Ae*. *simpsoni*, 1,259 *An*. *gambiae s*.*l*., 56 *An*. *funestus s*.*l*., 1 *An*. *coustani*, 37,777 *Culex* spp., 8 *Mansonia* spp. and 3 *Toxorhynchites* spp.

## Discussion

There is high possibility that the abundance and distribution of *Ae*. *aegypti* may increase in Africa as this continent is undergoing a rapid urbanization with a projection that over half of its population will be living in urban areas by 2050 [34]. The finding that more *Ae*. *aegypti* adult mosquitoes were collected in urban than rural areas is consistent with the adaptation of this species to the domestic environment as its abundance is positively correlated with increasing urbanization [6,35–38]. The fact that Africa’s urbanization is occurring at low levels of income and with far less infrastructural development, notably unreliable water supply and disposal of solid container wastes [39], suggest that the spread of *Ae*. *aegypti* may be greatly enhanced in future years.

The collection of more *Ae*. *aegypti* adult mosquitoes outdoors than indoors in all the four sites is consistent with other studies in Kenya [16,40], in Trinidad [41], in Malaysia [36,42] and in Brazil [43]. This is mainly because it has adapted to breed in a wide range of artificial containers that are mostly located outdoors around human dwellings [44,45]. Water storage containers constitute the main *Ae*. *aegypti* breeding habitats, especially those that remain undisturbed for several days [46]. This is usually a response by residents to unreliable rainfall and water supplies. Another adaptation is that female *Ae*. *aegypti* mosquitoes have developed a preference for human blood over that of other animals [47,48]. Hence, readily available breeding habitats and blood meal sources within the human surroundings makes *Ae*. *aegypti* adult mosquitoes to disperse short distances [10,11].

*Aedes aegypti* mosquitoes are known to bite during the daytime hours. However, their blood meal seeking activities were found by HLC to be highest in the morning and afternoon hours. This bimodal blood meal seeking behavior is similar to the findings obtained in Trinidad by Chadee [49]. Significantly more *Ae*. *aegypti* mosquitoes were collected in the afternoon than morning hours indicating the possibility that most of the human-vector contact is occurring in the afternoon hours. This finding is consistent with the experimental results by Gouck and Smith [50] but inconsistent with the findings by Strauss and others [51] who found no significant difference in the number of *Ae*. *aegypti* mosquitoes that fed at different times of the day. High multiple-feeding rates on humans and the ability to feed on community visitors, especially those visiting in the afternoon hours, makes *Ae*. *aegypti* an efficient vector to transmit and rapidly spread arboviral diseases within and among villages [12].

These *Ae*. *aegypti* adaptations have major implications for the possible transmission of diseases and for the planning of surveillance and control programs. For instance, identifying areas with daytime *Ae*. *aegypti* biting activity can cause one to be equipped with personal protective measures like repellents [52–55] in order to prevent or minimize chances of being bitten. If not very necessary, visiting such places can be avoided at all [55]. Public places, such as schools, where children spend much of the daytime can be prioritized for surveillance and for control measures which may include larval source reduction and fumigation [55,56]. Fumigation can be planned to coincide with peaks in landing periodicity of the *Ae*. *aegypti* adults in the morning and afternoon [49], but most preferably in the afternoon to evening hours. Evidence supports cleaning up the environment to be effective to prevent the spread of arboviral disease by *Ae*. *aegypti* [57,58]. Such clean-up activities can be maintained year-round in order to keep *Ae*. *aegypti* mosquitoes under control. Surveillance teams can target areas outside the home to monitor whether they may be possible sources of disease outbreaks. It is important to note that currently there are no any control methods against *Ae*. *aegypti* mosquitoes done by either the national or county governments in Kenya. However, some individuals may be using mainly commercially available aerosols to control these mosquitoes at household levels.

A number of limitations were noted during the implementation of this study. Only one BG trap was set per site as its cost was limiting to acquire more. HLC sampling was conducted from 5:30 am to 11:30 am and then from 3:30 pm to 7:30 pm. There is a possibility that *Ae*. *aegypti* adult mosquitoes were missed before 5:30 am, between 11:30 am and 3:30 pm and after 7:30 pm. Further identification of *Ae*. *aegypti* mosquitoes to either as *Ae*. *aegypti aegypti* or *Ae*. *aegypti formosus* could not be logistically performed within the scope of this study.

In conclusion, most of the *Ae*. *aegypti* adult mosquitoes were collected in urban areas, outdoors and in the afternoon hours in our western and coastal Kenya sites. These *Ae*. *aegypti* characteristics have major implications for the possible transmission of arboviral diseases and for the planning of surveillance and control programs.23

## Supporting information

S1 Database This database contains a description of codes, *Aedes aegypti* human landing catches and rainfall data, *Aedes aegypti* Prokopack automated aspirator and rainfall data, *Aedes aegypti* Biogents-sentinel traps and rainfall data, *Aedes aegypti* Prokopack automated aspirator blood-feeding stages data and other mosquito species data used. (XLSX)
